# Role of Fluid Milk in Attenuating Postprandial Hyperglycemia and Hypertriglyceridemia

**DOI:** 10.3390/nu12123806

**Published:** 2020-12-11

**Authors:** Miriam Leary, Hirofumi Tanaka

**Affiliations:** 1 Division of Exercise Physiology, Department of Human Performance and Applied Exercise Science, School of Medicine, West Virginia University, P.O. Box 9225, Morgantown, WV 26506-9225, USA; Miriam.leary@hsc.wvu.edu; 2 Cardiovascular Aging Research Laboratory, Department of Kinesiology and Health Education, The University of Texas at Austin, Austin, TX 78712, USA

**Keywords:** milk, dairy, postprandial metabolism, hyperglycemia, hypertriglyceridemia

## Abstract

Postprandial plasma glucose and triglyceride concentrations are predictive of relative cardiovascular disease (CVD) risk, and the pathogenesis of both insulin resistance and atherosclerosis has been attributed to acute states of hyperglycemia and hypertriglyceridemia. Postprandial lipemia and hyperglycemia suppress vascular reactivity and induce endothelial dysfunction. Epidemiological studies suggest that chronically-high consumption of milk and milk products is associated with a reduced risk of type 2 diabetes, metabolic syndrome, and CVD. The addition of dairy products to meals high in carbohydrates and fat may lessen these risks through reductions in postprandial glucose and triglyceride responses. Purported mechanisms include dairy proteins and bioactive compounds, which may explain the inverse relationship between dairy consumption and cardiometabolic diseases. The current review evaluates the available literature describing the relationships between metabolic dysfunction, postprandial metabolism, and vascular dysfunction and discusses the potential role of milk and dairy products in attenuating these impairments.

## 1. Introduction

Traditionally, fasting plasma glucose and triglyceride concentrations have been used as clinical determinants of CVD (cardiovascular disease). However, as most individuals spend the majority of their awake and ambulatory time in a postprandial state, acute responses in plasma glucose and triglycerides following a meal have been shown to be better predictors of relative CVD risk than fasting measures [1,2,3]. Epidemiological data indicate that two-hour glucose concentration measured during an oral glucose tolerance test (OGTT) is an independent predictor of CVD risk while fasting glucose concentration is not [4]. Similarly, postprandial triglycerides are better predictors of atherosclerosis and coronary artery disease (CAD) than fasting concentrations [5,6].

The pathogenesis of both insulin resistance and atherosclerosis has been attributed to acute states of hyperglycemia and hypertriglyceridemia. Indeed, postprandial hyperglycemia and hypertriglyceridemia are associated with increased risk of CVD and mortality [4,6,7,8]. Elevated postprandial plasma glucose or triglyceride concentrations following a high-carbohydrate or high-fat meal contribute to a pro-atherogenic metabolic state [9,10,11]. Moreover, both postprandial lipemia and hyperglycemia are associated with reduced vascular reactivity and impaired endothelial function [12,13].

**Figure 1:**
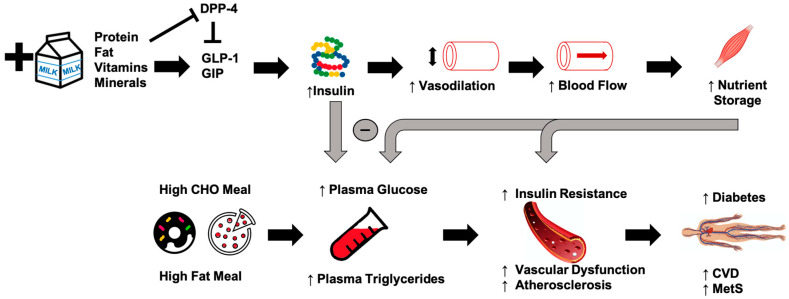
The addition of milk and dairy products to a meal high in carbohydrates or fat may potentially lessen the risk of cardiometabolic diseases through reductions in postprandial reductions in blood glucose and triglycerides. Purported mechanisms include dairy proteins and bioactive compounds acting via vascular function. CHO = carbohydrate, CVD = cardiovascular disease, MetS = metabolic syndrome, GIP = glucose-dependent insulinotropic polypeptide, GLP-1 = glucagon-like-peptide-1, DPP-4 = dipeptidyl peptidase IV.

Epidemiological studies suggest that chronically-high consumption of milk and milk products is associated with a reduced risk of type 2 diabetes [14,15,16], metabolic syndrome [17], and CVD [18]. The addition of dairy products to a meal high in carbohydrates or fat may potentially lessen the risk of cardiometabolic diseases through reductions in postprandial glucose and triglycerides [19,20,21] (Figure 1). Potential mechanisms driving this could include the proteins or bioactive compounds found in dairy. For instance, several of dairy’s bioactive compounds possess functional properties although the identification of the exact component responsible for reduced postprandial glycemia/lipemia is difficult to ascertain. Despite their low glycemic index, milk products, specifically milk proteins, are insulinotrophic, which may be a potential mechanism to explain, at least in part, the inverse relationship between dairy consumption and cardiometabolic diseases [19,22]. Insulin receptors are found on endothelial and vascular smooth muscle cells, and binding initiates vasodilation [23]. When consumed with a meal, dairy proteins generate a hyperinsulinemic response that may aid in postprandial vasodilation [24], thereby increasing limb blood flow and capillary recruitment for nutrient disposal [25,26], and facilitate the clearance and storage of glucose and triglycerides after a meal [27,28,29]. The resultant increase in clearance of glucose and triglycerides may lead to an attenuated impairment of postprandial vascular dysfunction. The focus of the current review is to evaluate the available literature describing the relationships between metabolic dysfunction, postprandial metabolism, and vascular dysfunction, and to discuss the potential role of milk in attenuating these impairments.

## 2. Postprandial Metabolism and Acute Vascular Dysfunction

In the past, clinical evaluation of hyperglycemia and hypertriglyceridemia as a predictor of CVD and mortality has utilized fasting measures. In more recent years, it is becoming increasingly well accepted that atherosclerosis is a postprandial phenomenon [30]. Most humans eat every four to five hours but require upwards of eight hours for complete clearance of glucose and triglycerides from the blood stream [31]. This suggests that most humans spend the majority of their day in a postprandial state. Therefore, postprandial measures represent a more robust indication of daily plasma glucose and triglyceride exposure and are a truer reflection of the associated risks [1,4,6,11].

### 2.1. Hyperglycemia

Precise matching of glucose utilization with endogenous glucose production and dietary glucose delivery is required for maintenance of normal plasma glucose concentrations. In a fasting state, plasma glucose concentration is maintained relatively stable as a tightly-regulated physiological variable [32]. After a meal, plasma glucose concentrations increase rapidly as glucose absorption increases to more than twice the rate of endogenous glucose production. Postprandial hyperglycemia depends on a number of factors including timing, quantity, and composition of the meal, the total amount of carbohydrate, the rate and degree of glucose absorption, the secretion of insulin, and inhibition of glucagon [33]. Epidemiological studies have consistently shown that plasma glucose levels two hours after an oral glucose challenge are significant predictors of CVD risk [7].

An intervention study using participants with impaired glucose tolerance determined whether an intervention to limit postprandial hyperglycemia would reduce the risk of CVD [34]. Acarbose, an α-glucosidase inhibitor that specifically reduces glucose absorption and thus postprandial hyperglycemia, was associated with a 34% reduction in risk of developing new cases of hypertension and a 49% risk reduction in CV events [34]. In addition, acarbose treatment is associated with a reduction in intima-media thickness in patients with type 2 diabetes [35] and a significant reduction in cardiovascular events independent of other risk factors [36]. In the progression of atherosclerosis, an LDL molecule enters the sub-endothelial space and becomes oxidized. After a meal, LDL oxidation increases acutely and augments the degree of hyperglycemia [37,38]. Clearly, attenuating postprandial hyperglycemia may positively influence CVD development.

The response of insulin following a meal is highly variable and depends on a number of factors, including timing, quantity, and composition of the meal, as well as the rate and degree of glucose absorption. The postprandial insulin response is also attributed to the secretion of insulinotropic amino acids as well as enterogastric incretin hormones (e.g., glucose-dependent insulinotropic polypeptide or GIP, and glucagon-like-peptide-1 or GLP-1). GLP-1 and GIP are secreted from enteroendocrine cells in a nutrient-dependent manner and their release stimulates glucose-dependent insulin secretion via binding to their distinct receptors on the pancreatic β-cells to stimulate insulin secretion. Both incretins promote expansion of β cell mass by stimulating β cell proliferation and inhibiting apoptosis [39]. GIP and GLP-1 are metabolized by dipeptidyl peptidase IV (DPP-4), which is thought to assist in the retention of incretin-related insulinotropic activity. In addition to incretins, recent in vitro data in preadipocytes have shown that certain proteases (e.g., membrane metallo-endopeptidase/neprislyin) favor insulin signaling and may enhance insulin sensitivity [40].

### 2.2. Hyperglycemia and Vasculature

Acute hyperglycemia, independent of insulin levels, significantly attenuates forearm endothelium-dependent, but not independent, vasodilation in healthy humans [13]. In both diabetic and healthy adults, hyperglycemic spikes have been shown to induce endothelial dysfunction [33,41]. It is purported that these effects are linked with a reduced bioavailability of nitric oxide (NO) since the hyperglycemia-induced endothelial dysfunction is counterbalanced by arginine [41]. One mechanism proposed is that hyperglycemia activates reactive oxygen species production that disrupts endothelial tight junctions, inducing endothelial permeability [42]. The rapid decrease in brachial artery flow-mediated dilation (FMD), a measure of endothelium-dependent vasodilation, is inversely correlated with the magnitude of postprandial hyperglycemia in patients with type 2 diabetes [43].

There are a number of other ways that hyperglycemia could influence vasculature. Thrombin is a molecule involved in the coagulation cascade, alterations of which are linked to thrombosis [44]. Postprandial hyperglycemia causes an overproduction of thrombin, which has been shown to be strictly dependent on blood glucose levels [44]. Further, adhesion molecules regulate the interaction between endothelium and leukocytes, and intracellular adhesion molecule-1 (ICAM-1) is increased in patients with type 2 diabetes and/or vascular disease. Postprandial hyperglycemia has been shown to be a sufficient stimulus for increased circulating levels of ICAM-1, thus activating one of the first stages of atherosclerosis [45,46]. Finally, acute hyperglycemia has been shown to increase the production of inflammatory cytokines [47,48]. These increases in thrombin, intracellular adhesion molecules, and inflammatory cytokines in the postprandial state could contribute to the impaired vascular function observed during acute hyperglycemia.

### 2.3. Hypertriglyceridemia

Lipoprotein lipase (LPL), present on the luminal side of endothelial cells, is responsible for the hydrolysis of triglycerides into glycerol and free fatty acids. After consumption of a high-fat meal, chylomicrons carry triglyceride through the vasculature where fatty acids are liberated by lipoprotein lipase and taken up by surrounding adipose and muscle cells [49]. Triglyceride removal is dependent on LPL activity and the tissues’ need for lipids [50]. In a postprandial state, LPL availability becomes limited due to competition for binding sites and causes triglyceride-rich lipoproteins such as chylomicrons to accumulate [9,51].

The magnitude of postprandial plasma lipid increase is directly proportional to the fat content in meals [10,52,53]. After a high-fat meal, plasma triglycerides increase, peaking around four hours and returning to fasting levels around eight hours [31]. Because a fatty meal increases plasma lipids, multiple high-fat meals throughout the day results in prolonged presence of elevated plasma triglyceride. Increased plasma triglyceride results in reduced HDL cholesterol and increased small, dense LDL. This contributes to an increased susceptibility to oxidation and a perpetual cycle of hypertriglyceridemia and atherosclerosis [54]. Postprandial triglyceride concentrations are associated with carotid artery wall thickness as measured by the intima-media thickness [55,56,57] and are better predictors of atherosclerosis and coronary artery disease than fasting levels [2,58]. Every 1.1 mmol/L increase in postprandial triglyceride concentration is associated with a 1.4 increase in relative risk for a myocardial infarction [59]. The classification based on the magnitude of postprandial hypertriglyceridemia demonstrates a 68% accuracy in detecting the presence of CVD [6]. Importantly, different metabolic effects and risks are associated with various types of fat (saturated vs. unsaturated fat) as well as the specific fatty acids consumed [60].

### 2.4. Hypertriglyceridemia and Vasculature

Endothelial dysfunction is present in states of hypertriglyceridemia and hypercholesterolemia and is attributed in large part to reduced bioavailability of NO [61]. Though the exact mechanism for the reduced NO bioavailability is unknown, it could involve any number of impairments to receptors, L-arginine use, concentration and activity of endothelial NO synthase, release and diffusion of NO, and oxidative inactivation of NO by superoxide [62]. High levels of postprandial plasma triglycerides after a high-fat meal are associated with endothelial dysfunction [63]. Indeed, a high-fat meal transiently impairs endothelial function, and mean triglyceride changes are proportionally associated with endothelial dysfunction [64]. Moreover, the infusion of a triglyceride emulsion induces a loss of vascular reactivity [65]. Even in young healthy adults, postprandial triglyceride levels are closely associated with impaired brachial artery FMD after a high-fat meal [66].

Acute hypertriglyceridemia induced by a high-fat meal correlates positively with changes in leukocyte production of reactive oxygen species. These changes are not seen with a low-fat meal, indicating that acute hypertriglyceridemia causes endothelial dysfunction via enhanced oxidative stress that reduces NO bioavailability [67]. Hypercholesterolemia impairs the L-arginine pathway, through which NO is produced, by activating the angiotensin-II receptors to cause vasoconstriction and neurohumoral activation. This facilitates the release of reactive oxygen species thereby further decreasing NO bioavailability and increasing vascular cell apoptosis and expression of adhesion molecules, chemotactic factors, and pro-inflammatory cytokines [68]. These cellular mechanisms likely contribute to vascular impairment observed during postprandial hypertriglyceridemic states.

## 3. Milk and Dairy

Considering that for most individuals in modern society, a majority of time is spent in a postprandial state, identification of treatments that moderate or attenuate postprandial hyperglycemia and hypertriglyceridemia is critically needed. Lifestyle modifications, including dietary interventions, offer an affordable and easily implemented alternative to pharmacological interventions.

Bovine milk is comprised of approximately 87% water, 4–5% lactose, 3% protein, 3–4% fat and less than 1% of vitamins and minerals combined. Milk supplies 32 g of protein per liter. Of the milk protein fraction, 20% is whey, a soluble protein, and 80% is casein, an insoluble protein. Both are considered high-quality proteins because they provide essential amino acids, are readily digested, and have high bioavailability. These two protein fractions differ in their amino acid profile. Whey protein is rich in branched chain amino acids whereas casein is higher in histidine and phenylalanine. The fat fraction of milk, present as globules, is dependent on animal origin, stage of lactation, and feed-related factors. Typically, the fat found in milk is comprised of 98% triglycerides, of which 70% is saturated fatty acids (e.g., palmitic acid, myristic acid, stearic acid, butyric acid) and 30% is unsaturated fatty acids (e.g., oleic acid, linoleic acid, α-linoleic acid). Lactose, the carbohydrate found in milk, is a disaccharide sugar comprised of galactose and glucose. The glycemic index of lactose is 45 compared with the reference 100 for glucose. In addition to the primary macronutrient composition, dairy products have a specific micronutrient composition including calcium, magnesium, and vitamin D [69].

### Epidemiological and Prospective Studies on Dairy Intake

Both epidemiological and prospective studies indicate that chronic consumption of dairy exhibits a protective effect in preclinical populations. A diet including high consumption of dairy products show lower chances of having prediabetes, undetected diabetes, or prevalent diabetes, compared with a Western diet [70]. For individuals with prediabetes, a diet that includes dairy decreases postprandial glucose, insulin, and triglyceride response compared with a red meat/refined carbohydrate diet [71]. Total dairy intake is associated with lower odds of hyperglycemia over a 9-year follow-up in a prospective study of individuals with metabolic syndrome [72]. Findings from the Framingham Heart Study show an inverse correlation between dairy consumption and risk of incident prediabetes [73]. Total, low-fat, and high-fat dairy intakes are associated with 39%, 32%, and 25% lower risks of prediabetes whereas neither cheese nor cream and butter is associated with prediabetes. Additionally, only high-fat dairy and cheese exhibit a dose–response inverse association with incident type 2 diabetes. These findings suggest that incident prediabetes may vary by dairy product and type and by baseline glycemic status [73].

Beyond preclinical states, chronic dairy consumption is associated with a reduced risk of type 2 diabetes and CVD [14,15,16,74]. Specifically, for each daily serving of dairy consumed, there were 9% and 4% decreases in risk of type 2 diabetes in men and women 14-16. Additionally, milk consumption is inversely associated with the overall risk of CVD and stroke [75,76]. There is a 15% lower relative risk for all-cause mortality and an 8% lower overall relative risk of ischemic heart disease with high dairy consumption [74]. Similarly, there is an inverse association between dairy intake and metabolic syndrome development in healthy, overweight, and obese individuals [17,77]. Thus, the available evidence supports the benefits of dairy intake on cardiometabolic diseases.

## 4. Dairy and Glycemia

### 4.1. Dietary Intervention Studies

Dietary intervention studies investigating the effects of milk or dairy products and glucose response indicate a wide range of effects, likely due to the different types of dairy utilized. In a six-week randomized cross-over trial, participants replaced 13% of their daily energy intake with either butter or cheese of equivalent fat content [78]. Compared with the butter trial, fasting blood glucose concentration increased after the cheese intervention although insulin resistance values were not different between the trials [78]. An eight-week clinical trial investigating the effects of low-fat dairy intake (low-fat milk and yogurt) on overweight and obese men found no effect on fasting glucose concentration [79]. However, a six-week randomized control trial found decreased plasma glucose concentrations with high dairy consumption compared with a control food in obese women [80]. Fasting blood glucose concentration did not change within or between trials in patients with hypertension who consumed 4+ servings of low-fat dairy or eliminated all dairy for 4 weeks [81]. As all studies used low-fat dairy as an intervention, the discrepancies are likely attributed to metabolic health or, more specifically, glycemic states of the population studied.

### 4.2. Postprandial Intervention Studies

The effects of dairy on postprandial metabolism indicate that milk elicits favorable effects on glucose metabolism both alone or with a meal. In a well-designed randomized cross-over study [82], whole milk (a control), a beverage based on equivalent milk macronutrients, complete milk protein (16 g), lactose (24 g), or milk fat (16 g) were compared. Whole and simulated milk lowered blood glucose concentration more than predicted by the sum of individual dietary components 83. Low-fat milk reduced post-meal peak blood glucose concentration and post-meal glucose area under the curve (AUC) compared with water, soy beverage, 1% chocolate milk, orange juice, or a cow milk-based infant formula [83,84].

The majority of intervention studies examining postprandial metabolism and dairy employed milk-derived proteins, specifically whey protein. The incremental AUC for glucose decreased in a dose-dependent manner with the highest dose of whey protein supplement having a significantly greater effect than lower doses on postprandial hyperglycemia from a glucose drink [85]. Similarly, increasing doses of whey protein (10–40 g) pre-meal reduced post-meal blood glucose and insulin AUC in a dose-dependent manner [86]. The combination of whey protein and carbohydrate intake results in increased plasma insulin and reduced plasma glucose concentrations compared with those consuming carbohydrate alone [87]. The addition of whey protein to a high glycemic meal for breakfast and lunch increases plasma insulin concentration by 31% at breakfast and 57% at lunch compared with meals without whey. Further, the consumption of whey decreases postprandial plasma glucose concentration by 21% compared with the meal without whey [27]. A study on individuals with type 2 diabetes who consumed 50 g of whey or placebo with a high glycemic breakfast found that glucose levels were reduced 28% and insulin increased 105% after the protein preload [88]. Interestingly, while not compared in a head-to-head fashion, the decrease in glycemia was a larger reduction than that observed after different doses of a rapid-acting non-sulfonylurea insulin secretagogue [88,89]. Taken together, whey protein consumption both in healthy and diabetic individuals appears to attenuate the rise in postprandial glycemia when combined with a high-carbohydrate load.

Dairy products appear to have favorable effects on post-consumption glycemia independent of insulin [90]. Milk products give rise to insulinemic responses far exceeding expected insulin production independent of their lactose production, suggesting that some component of milk is responsible for stimulating insulin release [22]. Independent of a direct effect on insulin, dairy’s bioactive compounds, in particular whey amino acids, appear to act on incretin hormones via bioactive peptides and amino acids released during digestion. Several gut hormones are stimulated (e.g., cholecystokinin, peptide YY) that increase insulin secretion while others serve as endogenous inhibitors (e.g., dipeptidyl peptidase-4) preventing incretin degradation [88].

## 5. Dairy and Lipemia

The effects of regular dairy consumption on lipid profiles has been examined in the literature [91,92,93]. While increased consumption of saturated fatty acids is associated with increased LDL cholesterol concentration, a number of intervention studies using whole milk and other whole fat dairy products have not shown significant increases in LDL cholesterol [91,92,93]. For lipid profiles, a higher proportion of small dense LDL (sdLDL) particles represents a greater atherogenic risk than larger, less dense LDL cholesterol molecules. In a cross-sectional study of healthy men, sdLDL particles were positively associated with plasma triglycerides and fasting insulin levels and inversely associated with HDL cholesterol concentrations. Individual fatty acids typically found in milk products (e.g., palmitic acid, myristic acid, butyric acid) are associated with fewer sdLDL particles, suggesting that milk’s fatty acids are associated with a more favorable lipid profile [94].

### 5.1. Dietary Intervention Studies

In a 10-year longitudinal study, a higher intake of dairy saturated fatty acids was associated with a lower CVD risk compared with a higher intake of saturated fat from meat products [60]. Interestingly, substitution of 2% of energy from saturated fat from meat with energy from dairy-derived saturated fat was associated with a 25% lower CVD risk. This attenuation has been attributed to other components of dairy such as calcium, magnesium, and/or bioactive peptides as well as to the relative proportions of different saturated fatty acids in meat and dairy [60]. However, a five-year prospective study of 300 women demonstrated that total dairy, milk, yogurt, cottage cheese, and calcium were positively related to triglycerides and negatively to HDL cholesterol at baseline, but no association was found for any five-year changes [95]. Moreover, healthy normocholesterolemic males who consumed 20% of dietary energy as butter for 21 days showed no significant change in blood lipid or apolipoprotein profile [93].

The available studies show conflicting results regarding milk protein’s effects on lipid profiles. Whey protein isolate supplementation over 3 months reduced fasting triglycerides, total cholesterol, and LDL cholesterol concentrations in overweight and obese adults [96]. A similarly-designed study employing a malleable protein matrix (protein-enriched yogurt) reduced fasting triglycerides, with the effect more pronounced in those with elevated triglycerides at baseline [97]. In contrast, three-month supplementation with whey protein during a weight regain study showed no effect on plasma lipids [98]. In a study examining the effects of lactotripeptide supplementation with or without physical exercise on vascular measures, there was no change in lipid panel including total cholesterol, LDL, HDL, or triglycerides in postmenopausal women after eight weeks of lactotripeptide supplementation [99]. Thus, the available evidence indicates that milk proteins may have a beneficial effect at least in some individuals (e.g., postmenopausal women).

### 5.2. Postprandial Intervention Studies

Postprandial triglyceridemia is strongly influenced by the composition of the meal, including the quality and quantity of fat. To date, there are few studies examining the role of complete dairy products on postprandial metabolism [20,21,100]. As such, the majority of dairy and lipemia studies have examined the effects of milk-derived proteins on postprandial metabolism. The postprandial appearance of triglycerides decreased by 21% and 27% when a meal was consumed with whey and casein, respectively [101]. There was no difference between four milk-derived proteins (α-lactalbumin, whey isolate, caseinoglycomacropeptide, and whey hydrolysate) on postprandial plasma triglycerides during an eight-hour high-fat test [102]. Postprandial apolipoprotein B-48 (apo B-48) response to a high-fat meal was significantly reduced when consumed with 60 g of whey protein compared with an equivalent dose of casein protein, independent of medium-chain saturated fatty acids. This reduced apo-B-48 is indicative of a reduced number of chylomicron particles within the blood stream, suggesting the potential of dairy to reduce the CVD risk associated with a high-fat meal [103]

## 6. Vascular Function and Dairy

In general, intervention studies examining the effects of dairy intake suggest favorable effects on vascular function. A recent scoping review reported that proteins derived from milk are common interventions for prediabetes and that significant improvements in brachial artery flow-mediated dilation and oxidative stress markers are observed without any evidence to support the benefit of milk proteins on insulin or lipid profiles [104]. In the early stages of atherosclerosis, damage to vessel walls can be protected by milk-derived bioactive peptides, which have anti-inflammatory effects on endothelial cells [105]. Additionally, bioactive peptides from milk have been found to behave as angiotensin converting enzyme-inhibitors, anti-thrombotics, and anti-oxidants, all of which favorably affect the vasculature [106,107,108].

In a two-month study examining the effects of lactotripeptide supplementation (isolated from sour milk) with or without exercise on vascular functions in postmenopausal women, lactotripeptide supplementation, alone and with concomitant aerobic exercise, reduced arterial blood pressure, improved a measure of endothelium-dependent vasodilation, and reduced central artery stiffness [99,109]. A one-month study supplementing patients with essential hypertension with a specially formulated whey protein blend showed improvements in vascular reactivity as measured by flow-mediated dilation [110]. Participants who consumed both whey and casein with concomitant aerobic exercise demonstrated favorable changes in several arterial stiffness parameters [111].

Solitary addition of conventional non-fat dairy products to the routine diet reduces blood pressure and improves vascular function in middle-aged and older adults with elevated blood pressure [81,112]. However, similar dietary interventions using whole milk and full-fat dairy products do not appear to result in similar hypotensive or vascular enhancing effects [113,114]. These findings suggest that the effects of consuming an isolated dairy component when consumed as whole dairy foods depend on both the dairy component and the food itself.

Despite the beneficial effects of the bioactive peptides derived from milk proteins, only a limited number of research studies have been conducted investigating vascular effects during the postprandial state with milk or milk proteins. Previous studies in adults with prediabetes reported that dairy milk, presumably mediated by its protein content, attenuates postprandial hyperglycemia-induced impairments in vascular endothelial function by limiting oxidative stress [115]. These effects, in turn, act to improve nitric oxide bioavailability to the vascular endothelium by increasing arginine availability and limiting competitive inhibition on NO biosynthesis by asymmetric dimethylarginine [115]. Milk proteins have yet to demonstrate beneficial effects on arterial stiffness, as assessed by pulse wave velocity, in the postprandial state [101,116,117]. Another key measure of peripheral vascular function, flow-mediated dilation, a measure of endothelial-dependent vasodilation, may change acutely as it is depressed in hyperglycemic, hyperlipidemic, and hyperinsulinemic states [118]. In overweight adults with hypertension, FMD was 4% greater at 120 min with a whey protein derivative [119]. Additionally, low-fat milk maintains conduit vessel vascular endothelial function in adults with metabolic syndrome by limiting postprandial hyperglycemia when compared with rice milk [120]. In a placebo-controlled, randomized, crossover experimental study, overweight and obese participants with normal metabolic health exhibited no differences in metabolic and hemodynamic responses between non-fat milk and the control drinks [100]. However, when participants were divided into tertiles of dietary fat intake, non-fat milk attenuated acute hypertriglyceridemia in individuals who regularly consumed a high-fat diet. There were no differences in key vascular functions including FMD, femoral blood flow, and femoral vascular conductance between trials, suggesting that non-fat milk can manage elevated postprandial triglycerides in those at risk for CVD independent of hemodynamic changes [100].

The acute vascular effects of dairy in a postprandial setting have been minimally investigated, but the overall findings suggest a favorable response. The potential for dairy to limit postprandial impairments in FMD indicates significant therapeutic applications for at-risk individuals. However, the investigations into the vascular effects of dairy are still in an early stage and mechanisms of action have yet to be fully determined.

## 7. Mechanisms of Improvement Induced by Dairy Intake

### 7.1. Protein

Of the milk protein fraction, 20% is whey and 80% is casein. Whey and casein differ in their amino acid profile and while both are readily digestible, casein is digested more slowly. Whey protein is a soluble protein whereas casein, an insoluble protein, clots in the stomach causing a delay in gastric emptying and results in the slower release of amino acids through the gastrointestinal tract [121]. Whey and casein both stimulate incretins hormones (GIP, GLP-1), and their potency may be altered when proteins are consumed in a hydrolysate form. The differing effects of whey and casein proteins on incretins are described below and elsewhere [122,123]. The biologically active protein fragments from milk are released from parent proteins after enzymatic action. It is in this form that these functional amino acid sequences are able to exert effects on receptors or interact with enzymes to elicit effects on metabolism. For example, native whey protein, with a higher leucine content, modulates postprandial hyperglycemia, compared with a placebo [124].

The beneficial effects of milk, as seen in cross-sectional and epidemiological studies [14,15,16,17,74,75,76,77,91,92,93,94], are supported by clinical trials and postprandial intervention studies [20,21,78,79,80,81,82,83,84,100]. Regardless of the outcome, nearly all research studies attribute the favorable metabolic effects of dairy, at least in part, to the insulinotropic effects of milk proteins [27,85,86,87,88,101,103]. Indeed, compared with other protein sources, milk demonstrates a larger insulin response up to 4 h post-meal [19]. In general, milk proteins seem to exert greater effects on metabolic response in individuals with more disturbed metabolism, including preclinical and clinical populations.

The suggested mechanisms for improved glucose regulation include a protein-induced increase in insulin concentration when milk products are added to a meal high in carbohydrates. The high amino content, in particular branched chain amino acids (BCAA), may modulate glucose levels by increasing postprandial insulin secretion. Interestingly, whey hydrolysate elicits higher insulin responses compared with whole milk [125] and intact whey protein [126]. Likely, this is due to the partially digested proteins in whey hydrolysate eliciting a faster release of insulin.

The available studies investigating dairy-derived proteins suggest a cardioprotective effect in preclinical populations. For instance, proteins derived from milk are common interventions for prediabetes since significant improvements have been observed in oxidative stress markers and glucose metabolism, especially postprandial glucose and glycated hemoglobin A1c (HbA1c), without any improvements of insulin profiles [104]. A proprietary whey protein hydrolysate that contained bioactive peptides with α-glucosidase-inhibiting properties reduces postprandial hyperglycemia in prediabetes adults [124].

Whey protein reduces acute postprandial glucose response in healthy adults and those with type 2 diabetes [27,85,86,127,128]. Additionally, insulin-independent reduction in blood glucose is seen with whey protein consumption in healthy adults [128]. Chronic consumption of whey protein (55 g/day for 12 weeks) improves fasting insulin levels and homeostasis model assessment of insulin resistance (HOMA-IR) scores in obese individuals [96]. The insulinotropic effect of whey protein has been attributed to specific amino acids (more specifically, a mixture of leucine, isoleucine, valine, threonine, and lysine mimicking whey protein’s glycemic and insulinemic responses) 28 as well as activation of the incretin system in both healthy individuals [128,129] and those with type 2 diabetes [27]. Whey protein increases the postprandial GIP response compared with other dairy or protein sources although the effects of whey protein does not appear to be mediated by amino acids [28]. Postprandial effects of whey on GLP-1 is more equivocal as whey induces a rise in GLP-1 in healthy individuals [130,131], which is thought to be partly related to DPP-4 [132] (Figure 1). In other studies, however, GLP-1 did not change in response to whey ingestion in healthy [28,29] and type 2 diabetic adults [27].

The effect of casein on glucose metabolism is not as well studied as the effects of whey protein. While both whey and casein exhibit insulinotropic properties in healthy participants, whey is a more potent insulin secretagogue than casein [29]. In individuals with type 2 diabetes, casein hydrolysate (12 g) acutely increases the postprandial insulin response [133]. However, incretin hormones play a rather minor role in the insulinotropic effect of casein in adults with type 2 diabetes [134]. Interestingly, protein fractionation appears to drive differences in casein-mediated GIP, but not GLP-1, release [135].

Postprandial triglyceridemia is a characteristic of type 2 diabetes. But dairy proteins can modify these metabolic responses. For instance, compared with 20 g whey protein consumed with a high-fat meal, pre-meal protein intake (20 g whey protein) before a fat-rich meal increases hormone secretion (insulin, glucagon, and GIP) and delays gastric emptying, but does not influence lipid or glucose responses, in individuals with and without type 2 diabetes [136]. Compared with other protein sources (cod and gluten protein), whey protein isolate and casein lower the triglyceride response when consumed acutely with a high-fat meal in obese, non-diabetic adults [137]. Casein combined with carbohydrates and a high-fat meal suppress triglycerides response acutely in type 2 diabetes [134] in agreement with the observation that casein and whey protein have similar suppressive effects of postprandial triglyceride concentrations after a high-fat meal in non-diabetic adults [137]. Whey protein isolate lowers triglyceride response more than casein in postmenopausal women [138] while whey protein isolate consumed with a high-fat meal lowers triglyceride response more than casein in individuals with type 2 diabetes [101]. However, while proteins derived from milk are common interventions for prediabetes and exhibit positive outcomes on glucose metabolism, a recent review found no evidence to support the benefit of milk proteins to lipid profiles [104]. For additional reading substantiating the differences between whey and casein, the review by BjØrnshave and Hermansen is recommended [139].

A recent controlled, randomized clinical trial made an attempt to discern the role of milk protein in regulating postprandial hyperglycemia [140]. In individuals with the highest android obesity, non-fat milk attenuated acute hyperglycemia compared with a placebo drink matched for macronutrient and caloric content. Any differences in postprandial hyperglycemia between trials could not be attributed to the macronutrients, and specifically the protein quantity, of milk [140]. Additionally, the beneficial effects of dairy intake were associated with the elevated endothelium-dependent vasodilation [140]. These findings suggest that milk consumed with a high-carbohydrate meal may reduce hyperglycemic responses preferentially in pre-clinical individuals with less favorable cardiometabolic profiles and that these metabolic effects may be related to hemodynamic improvements.

### 7.2. Fat

While the protein content of dairy is typically the primary constituent associated with reductions in glycemia, evidence is emerging that points to dairy’s fat content as a potential contributor. In a cross-sectional study of Brazilian adults, the intake of total dairy was inversely associated with fasting glucose and postprandial glucose concentrations after adjusting for covariates. Interestingly, myristic acid (14:0), a long chain saturated fatty acid found in dairy foods, was the only apparent nutrient to mediate the association between dairy intake and glycemia, indicating that this saturated fatty acid may play an important role in improving glucose homeostasis [141]. This finding is supported by another study, which showed a positive relationship between myristic, palmitic, and stearic acid concentrations in plasma phospholipids and type 2 diabetes risks [142]. However, it is important to note that the glycemic control exerted by dairy products is evident in non-fat and low-fat dairy products. Thus, the potential role of milk fats in regulating glycemia is likely in addition to other mechanisms, such as the insulinotropic effects of proteins.

### 7.3. Vitamins and Minerals

In addition to the aforementioned potential mechanisms of protein and fat in altering metabolism, dairy also contains a number of bioactive compounds with functional properties. The combined vitamin and mineral contents comprise less than 1% of milk, but this rather small volume is capable of eliciting significant metabolic effects. A systematic review and meta-analysis found that vitamin D status, calcium, and dairy intake are all inversely associated with type 2 diabetes and metabolic syndrome [143]. This meta-analysis concludes that vitamin D and/or calcium supplementation play an important role in preventing type 2 diabetes in populations at high risk (i.e., glucose intolerance) [143].

The leading food source of vitamin D in the American diet is fortified cow milk, which contains ~100 IU/8 oz [144]. Evidence indicates that vitamin D may beneficially mediate metabolic syndrome risk factors [145]. In fact, serum vitamin D status is inversely related to metabolic syndrome, and higher serum vitamin D levels is associated with the reduced prevalence of metabolic syndrome by nearly 50% compared with lower vitamin D levels [146,147]. The suggested mechanisms for the effect include reduced dyslipidemia and increased insulin production [146]. Plasma triglyceride levels are lower in individuals with >92.5 nmol/L of vitamin D compared with those with <62.5 nmol/L [148]. Additionally, several intervention studies have shown improved glycemic responses with vitamin D treatment. However, these effects may be specific to individuals who were deficient at baseline or those who had preexisting metabolic disorders [149,150,151].

The high calcium content and lipid fractions in dairy may benefit the serum lipid profile. Nearly half of the calcium in the American diet is from dairy [144]. Several randomized control trials have shown beneficial effects from calcium supplementation on plasma lipids [152,153,154]. Compared with consuming their standard diet, participants who consumed 2200 mg/d of calcium from fortified foods for 10 days demonstrated decreased total cholesterol and LDL cholesterol concentrations [152]. Serum cholesterol concentration was reduced by 15 mg/dL and triglycerides reduced by 32 mg/dL with calcium supplementation of 900 mg/day. The proposed physiological mechanism is that calcium binding to saturated fatty acids within the intestines forms insoluble soaps that are excreted in the feces [155]. Healthy men consuming 1 L of milk or yogurt daily containing 1,200 mg calcium compared with placebo demonstrated increased fecal fatty acid and bile acid excretion during dairy intake [156].

Magnesium has also been proposed as a significant mediator in postprandial metabolism. Upwards of 10% of the magnesium in the American diet comes from dairy [69]. Data from epidemiological studies has consistently demonstrated an inverse relationship between dietary magnesium and risk for incident type 2 diabetes [157,158,159]. Clinical trials have demonstrated that oral magnesium supplementation improves insulin sensitivity, glucose homeostasis, and HbA1c levels in patients with type 2 diabetes [160]. Moreover, a meta-analysis of randomized control trials found 12 weeks of supplementing with magnesium significantly lowered fasting serum glucose in patients with type 2 diabetes [161]. These effects are generally attributed to improved insulin-elicited glucose uptake because magnesium is essential for optimal coupling and signaling through the insulin receptors. A recent review on magnesium and insulin resistance confirmed that magnesium supplementation assists in the control of insulin resistance in patients with hypomagenesemia [162]. Regarding dyslipidemia, magnesium has been shown to reduce serum triglycerides, apolipoprotein B, LDL cholesterol, total cholesterol, and to increase HDL cholesterol concentrations in patients with ischemic heart disease [163,164,165]. These effects have been attributed to modifications of several enzymes intricately linked with lipid metabolism [166].

Dairy’s micronutrient profile offers significant potential for favorable effects on the cardiometabolic profile. As with the insulinotropic effects of milk proteins, evidence suggests that these micronutrients offer their greatest benefits to individuals who are either deficient in the respective vitamin or mineral or who are farther along the spectrum of disease progression. However, their mechanisms of action have yet to be fully described as no study is yet to determine the role of dairy’s micronutrients in a controlled intervention trial.

## 8. Summary

The available literature describing the relationships between metabolic dysfunction, postprandial metabolism, and vascular dysfunction recognizes the role of milk, specifically milk proteins, in reducing these clinical risk factors. Milk and milk products are associated with a reduced risk of cardiometabolic diseases, and the addition of dairy products, in particular dairy proteins, to meals high in carbohydrates and fat may potentially lessen these risks through reductions in postprandial glucose and triglycerides. The mechanisms of these effects are attributed in part to the functional properties of several bioactive compounds found in dairy as well as the insulinotrophic properties of dairy proteins. Increased insulin production stimulates vasodilation which may serve to increase limb blood flow and capillary recruitment for nutrient disposal thereby facilitating the clearance and storage of glucose and triglycerides after a meal. The resultant clearance of glucose and triglycerides may lead to an attenuated impairment of postprandial vascular dysfunction and reduced risk of cardiometabolic diseases. The available literature suggests that the addition of milk may be an easily implemented means of reducing elevations in postprandial hyperglycemia and hypertriglyceridemia and resultant metabolic dysfunction from consuming a high-fat or high-carbohydrate meal.
